# Transcriptome Analysis Identifies Novel Genes Associated with Low-Temperature Seed Germination in Sweet Corn

**DOI:** 10.3390/plants12010159

**Published:** 2022-12-29

**Authors:** Yingni Xiao, Mei Chen, Nannan Zheng, Zhuoyi Xu, Jie Zhang, Xinmin Hu, Li Li, Riliang Gu, Xuemei Du, Jianhua Wang

**Affiliations:** 1 Beijing Innovation Center for Crop Seed Technology, Ministry of Agriculture and Rural Affairs, College of Agronomy and Biotechnology, China Agricultural University, Beijing 100193, China; 2 Guangdong Provincial Key Laboratory of Crop Genetic Improvement, Crops Research Institute, Guangdong Academy of Agricultural Sciences, Guangzhou 510640, China

**Keywords:** sweet corn, seed vigor, low temperature germination capacity, transcriptome sequencing

## Abstract

Typically, sweet corn, particularly *sh2* sweet corn, has low seed vigor owing to its high sugar and low starch content, which is a major problem in sweet corn production, particularly at low temperatures. There is considerable variation in the germination rates among sweet corn varieties under low-temperature conditions, and the underlying mechanisms behind this phenomenon remain unclear. In this study, we screened two inbred sweet corn lines (tolerant line L282 and sensitive line L693) differing in their low-temperature germination rates; while no difference was observed in their germination rates at normal temperatures. To identify the specifically induced genes influencing the germination capacity of sweet corn at low temperatures, a transcriptome analysis of the two lines was conducted at both normal and low temperatures. Compared to the lines at a normal temperature, 3926 and 1404 differently expressed genes (DEGs) were identified from L282 and L693, respectively, under low-temperature conditions. Of them, 830 DEGs were common DEGs (cDEGs) that were identified from both L282 and L693, which were majorly enriched in terms of microtubule-based processes, histone H3-K9 modification, single-organism cellular processes, and carbohydrate metabolic processes. In addition, 3096 special DEGs (sDEGs), with 2199 upregulated and 897 downregulated, were detected in the tolerant line L282, but not in the sensitive line L693. These sDEGs were primarily related to plasma membranes and oxygen-containing compounds. Furthermore, electric conductivity measurements demonstrated that the membrane of L282 experienced less damage, which is consistent with its strong tolerance at low temperatures. These results expand our understanding of the complex mechanisms involved in the cold germination of sweet corn and provide a set of candidate genes for further genetic analysis.

## 1. Introduction

Sweet corn is one of the most important vegetables grown worldwide [1,2,3] and a good source of certain minerals, vitamins, phytonutrients, and dietary fiber, which makes sweet corn an ideal crop for maintaining good health [4]. Recently, it was reported that China produces approximately 530,000 ha of sweet corn, thereby surpassing the United States as the top sweet corn producer.

Sweet corn is derived from mutations in genes involved in the starch biosynthesis pathway. Several genes that modify the carbohydrate composition of its endosperm have been revealed to show increased sugar content when mutated [5]. The *shrunken2* (*sh2*) gene encodes the large subunit of ADP-glucose pyrophosphorylase (AGPase), which is involved in the first rate-limiting step of the starch biosynthesis pathway [6]. The *sh2* mutants exhibit remarkable reductions in their total number of carbohydrates, resulting in a large increase in sugar content and a decrease in starch content [7]. Owing to their increased sugar content and long shelf life [8,9,10], the *sh2* mutants have become a revolutionary type of sweet corn and account for approximately 100% of the fresh market and 75% of the processing industry of sweet corn [3].

The low starch and high sugar content of the dry kernels of *sh2* sweet corn typically result in a reduction in energy reserves that is required for seed germination and seedling emergence [11,12]. Additionally, the *sh2* endosperm drastically shrinks during drying, and its pericarp becomes brittle, which makes the *sh2* seed susceptible to damage caused by environmental stress [1,13]. Hence, mature *sh2* seeds have poor germination and seedling vigor, particularly under extreme conditions, such as cold stress [1,11,14]. In northern China, the early sowing of maize has gradually gained popularity as a result of the occurrence of longer growing seasons in recent years. Sweet c10orn can be harvested in advance to obtain additional benefits via early sowing, which risks the occurrence of low-temperature stress during seedling emergence [1,15]. Therefore, the improvement of the plant’s cold tolerance during germination is crucial for sweet corn production.

Cold stress severely affects corn growth, typically causing damage to the membrane system, cell dehydration, the accumulation of reactive oxygen species (ROS), and protein denaturation [16]. In recent years, some low-temperature tolerance genes, such as *ZmMKK4*, *ZmICE1*, *ZmbZIP68*, *ZmRR1* and *ZmDREB1*, have been cloned from field corn via mutant analysis, QTL cloning, and comparative genomic analysis, which has increased the understanding of the genetic mechanism underlying low-temperature tolerance in maize [17,18,19,20]. However, there are few studies on the low-temperature tolerance of sweet corn, particularly during germination.

Transcriptome analysis is a powerful tool for identifying genes that contribute to complex traits, and several cold-responsive genes have been identified in maize [21]. Using transcriptome analysis, Mao et al. identified numerous differentially expressed genes (DEGs) related to sweet corn seedling growth under cold conditions [22]. However, little is known about sweet corn germination. In this study, we conducted a transcriptome analysis using two sweet corn lines, L282 and L693, with different germination resistances at low temperatures to explore the genes involved in the germination properties of sweet corn. The results will not only help to understand the difference in the low-temperature germination capacities between the two *sh2* sweet corn lines but also provide new candidate genes for breeding low-temperature-tolerant varieties of sweet corn.

## 2. Results

### 2.1. Inbred Line L282 Showed Better Germination Resistance to Low Temperature Than L693

**Figure 1:**
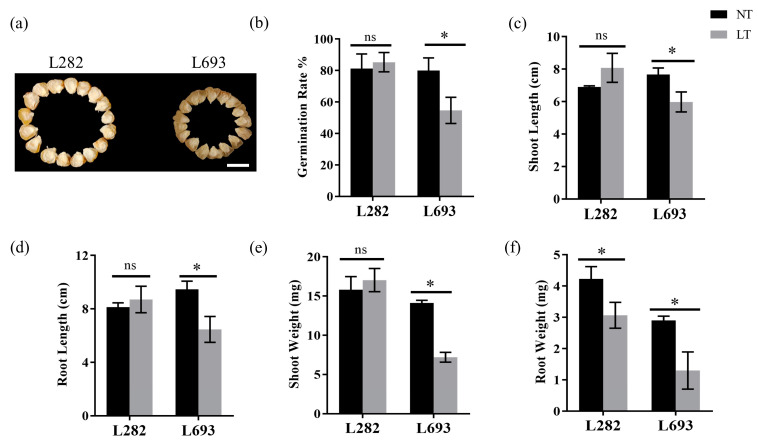
Characteristics of L282 and L693. (**a**) The mature dry seeds of the two lines, Bar = 1 cm; (**b**–**f**) comparisons of germination rate (**b**), shoot length (**c**), root length (**d**), shoot weight (**e**), and root weight (**f**) of the two lines germinated at normal temperature (NT) and low temperature (LT). The asterisks indicate significant difference at *p* < 0.05 (n = 3). The “ns” indicates no significant difference.

To test the low-temperature germination capacity of *sh2* sweet corn, two inbred lines, L282 and L693, were evaluated in terms of their germination rate (GR) in paper rolls (Figure 1a). At a normal temperature, both lines had similar GRs of about 80% (Figure 1b). At low temperatures, L282 showed a GR similar to that at normal temperature (Figure 1b); however, the GR of L693 reduced to 53% from a level of 80% for normal-temperature germination (Figure 1b). In addition, the root length (RL), shoot length (SL), and shoot weight (SW) of L282 were similar at normal and low temperatures (Figure 1c–e). Whereas L693′s RL, SW, and SL all reduced at low temperatures compared to normal temperature (Figure 1c–e). Root weight (RW) was reduced in both lines, but it decreased more significantly in L693 than in L282 (Figure 1f). These results indicate that L282 may have higher germination capacity at low temperature than L693.

### 2.2. Transcriptome Analysis of L282 and L693 Seeds Germinated at Normal and Low Temperatures

**Figure 2:**
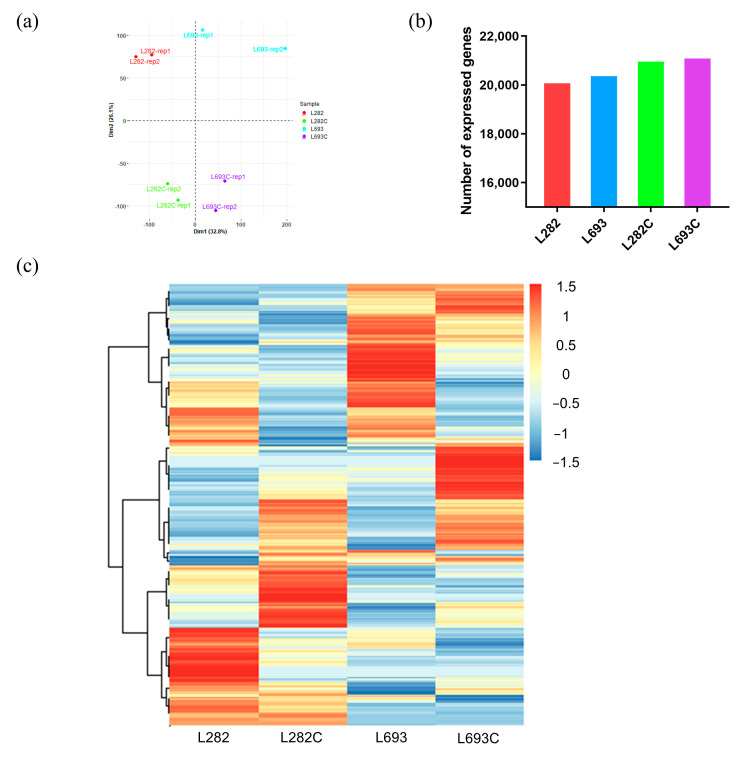
Transcriptome analysis of L282 and L693 seeds germinated at normal and low temperatures. (**a**) Principal component analysis of the gene expression profiles in L282 and L693 seeds germinated under normal (L282 and L693) and cold (L282C and L693C) conditions; (**b**) the number of expressed genes identified from L282, L693, L282C, and L693C; (**c**) heatmap clustering of the expressed genes. Red and blue indicate high and low abundances, respectively, according to the normalized FPKM.

To investigate the differences in the genes involved in germination between the two *sh2* sweet corn varieties L282 and L693, we performed a transcriptome analysis at normal and low temperatures. Eight RNA samples were prepared with two biological replicates from both lines that germinated under normal (L282 and L693) and cold (L282C and L693C) conditions. Our constructed principal component analysis (PCA) plots evidently separated the replicates of different lines and treatments, suggesting the reliability of our RNA-seq data (Figure 2a). Transcriptome sequencing revealed a total of 208,962,737 clean reads, with the reads for each sample ranging from 21,280,625 to 37,564,993 (Table 1).

After aligning the reads to the maize reference genome (Zm-B73-REFERENCE-G RAMENE-4.0), 88.19–90.11% (mean 89.11%) of the reads were mapped, with 73.93–80.55% (mean 78.19%) uniquely mapped (Table 1). The genes with fragments per kilobase of exon per million mapped fragments (FPKM) ≥1 were considered expressed genes, and the numbers of expressed genes in L282 (mean 20,172; range 19,998–20,346) and L693 (mean 20,460; range 20,143–20,778) were similar, which were fewer than those in L282C (mean 21,056; range 21,045–21,067) and L693C (mean 21,190; range 21,161–21,219) (Figure 2b, Table 1).

**Figure 3:**
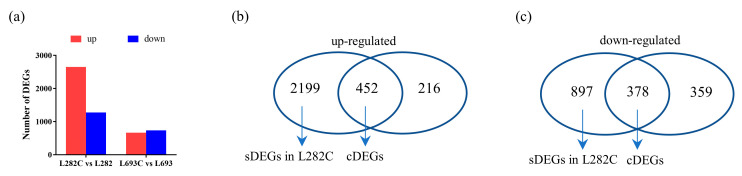
DEG analysis of L282 and L693 seeds germinated at normal and cold temperatures. (**a**) The numbers of upregulated and downregulated DEGs between L282C (L282 cold germination) and L282 (L282 normal germination) and those between L693C and L693; the common upregulated (**b**) and downregulated (**c**) DEGs (cDEGs) in both lines, and specially expressed DEGs (sDEGs) in L282C.

Hierarchical cluster analysis was performed to compare the gene expression patterns among the different samples, which showed great changes between the different lines and treatments (Figure 2c). Using the criteria of fold change ≥ 2 and false discovery rate (FDR) ≤ 0.05, the DEGs between L282C and L282 and those between L693C and L693 were defined. Compared to normal-temperature germination, low-temperature germination generated 3926 DEGs (2651 upregulated and 1275 downregulated) for L282 and 1404 DEGs (667 upregulated and 737 downregulated) for L693 (Figure 3a). These DEGs were considered cold-responsive genes. 

### 2.3. Identification of Common DEGs at Low Temperature

Of the identified cold germination-responsive DEGs, 830 (452 upregulated and 378 downregulated) were shared by L282 and L693, which were regarded as common DEGs (cDEGs) (Table S1) (Figure 3b,c). To further detect the functional pathways of the cDEGs, gene ontology (GO) analysis was performed using the AgriGO-v2 software (Table S2).

**Figure 4:**
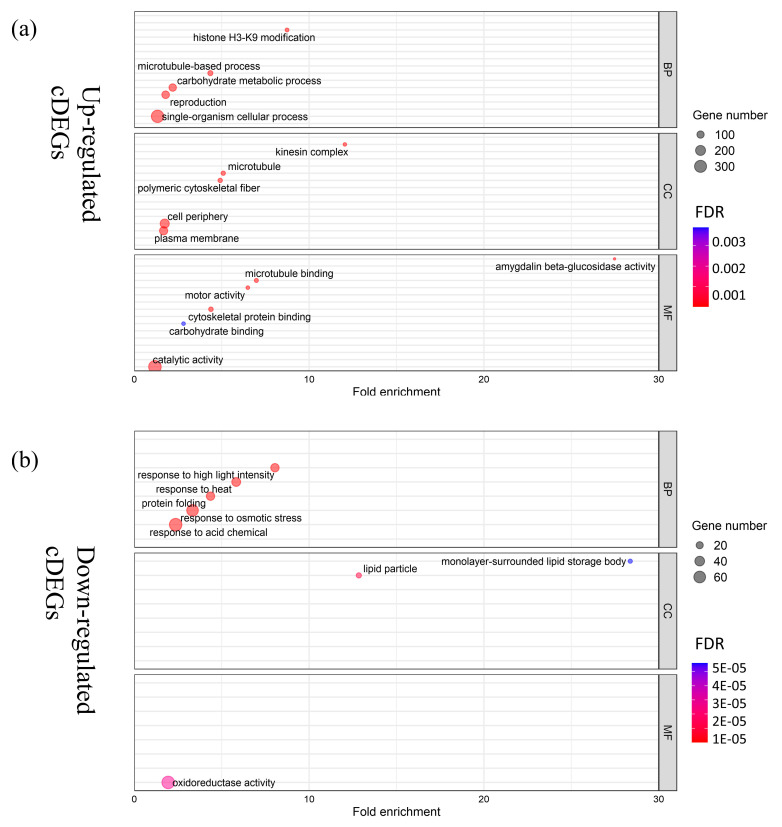
GO enrichment analysis of common DEGs (cDEGs) with upregulated (**a**) and downregulated (**b**) expressions. The size of the dots and color scale represent the number of cDEGs in the GO terms and the significance level (FDR), respectively.

Regarding the upregulated cDEGs, we found that the enriched biological process (BP) categories were primarily associated with microtubule-based processes, histone H3-K9 modification, single-organism cellular processes, and carbohydrate-metabolic processes (Figure 4a, Table S2). The significant molecular function (MF) categories were related to microtubule motor activity, cytoskeletal protein binding, motor activity, and amygdalin beta-glucosidase activity (Figure 4a, Table S2). The enriched cellular component (CC) categories were related to the cell periphery, the kinesin complex, polymeric cytoskeletal fiber, and microtubules (Figure 4a, Table S2).

For the 378 downregulated cDEGs, the significant categories were primarily related to the response to high light intensity, the response to heat, and the response to osmotic stress (Figure 4b, Table S2). These results showed that various processes, such as carbohydrate metabolism, the response to abiotic stimuli, histone H3-K9 modification, and single-organism cellular processes, can contribute to the low-temperature germination of both L282 and L693. 

### 2.4. Identification of L282 Specifically Expressed Cold-Responsive DEGs

**Figure 5:**
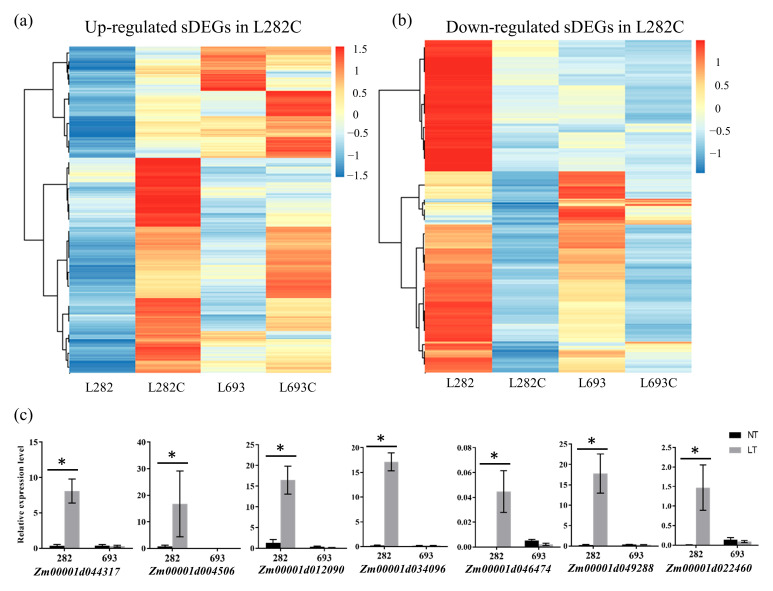
The analysis of L282′s specifically expressed DEGs (sDEGs) in response to low temperature. (**a**,**b**) Heatmap clustering of the upregulated sDEGs (**a**) and downregulated sDEGs (**b**). Red and blue indicate high and low abundance according to the normalized FPKM, respectively. (**c**) qRT-PCR analysis of seven sDEGs. The asterisks indicate significant difference at *p* < 0.05 (n = 3).

Compared to the sweet corn line L693, L282 showed strong tolerance to a low temperature (Figure 1). Of the identified cold germination-responsive DEGs, 3096 (2199 upregulated and 897 downregulated) were specifically identified from L282, but not from L693. These DEGs were considered L282 specifically expressed DEGs (sDEGs) (Figure 3b,c, Table S3). We compared the expression patterns of these sDEGs in L282 and L693 (Figure 5a,b) and found that they all showed dramatic changes in expression levels in L282 at low-temperature germination compared to normal-temperature germination, while in L693 they did not change in terms of expression patterns or change insufficiently (Figure 5a,b). To verify the reliability of gene expression characterized by transcriptome sequencing, the expression characteristics of seven sDEGs encoding heat shock protein, cytochrome P450, and pectin methylesterase were quantified using qRT-PCR. All genes showed similar changes to the RNA-seq results in L282, and in L693, they did not change significantly (Figure 5c, Table S3).

**Figure 6:**
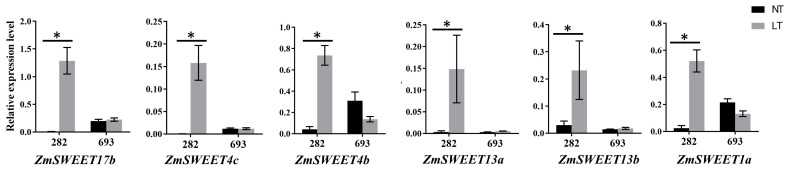
qRT-PCR analysis of six *ZmSWEET* genes expressed in L282 and L693 at normal temperature (NT) and low temperature (LT). The asterisks indicate significant difference at *p* < 0.05 (n = 3).

As the main characteristic of sweet corn is a change in carbohydrate composition, we focused on the sDEGs relating to carbohydrate metabolism and transport (Table 2). *Zm00001d025943* is a gene encoding fructofuranosidase, while *Zm00001d014866* encodes glycosyl transferase (Table 2). *Zm00001d031303* and *Zm00001d037480* are two genes encoding raffinose synthases, and *Zm00001d017502* and *Zm00001d029371* encode trehalose 6-phosphate phosphatase (Table 2). All six of these genes were significantly upregulated in the L282 seeds in response to low temperature, whereas their expression levels were not detected in the L693 seeds at both normal and low-temperature conditions (Table 2). Moreover, the expressions of eight sugar transporters*—ZmSWEET1a*, *ZmSWEET4a*, *ZmSWEET4b*, *ZmSWEET4c*, *ZmSWEET13a*, *ZmSWEET13b*, *ZmSWEET14a*, *ZmSWEET14b*, and *ZmSWEET17a*—were also significantly upregulated in L282C but were not detected in L693C (Table 2). The expression levels of six *ZmSWEET* genes were also confirmed by qRT-PCR analysis (Figure 6).

**Figure 7:**
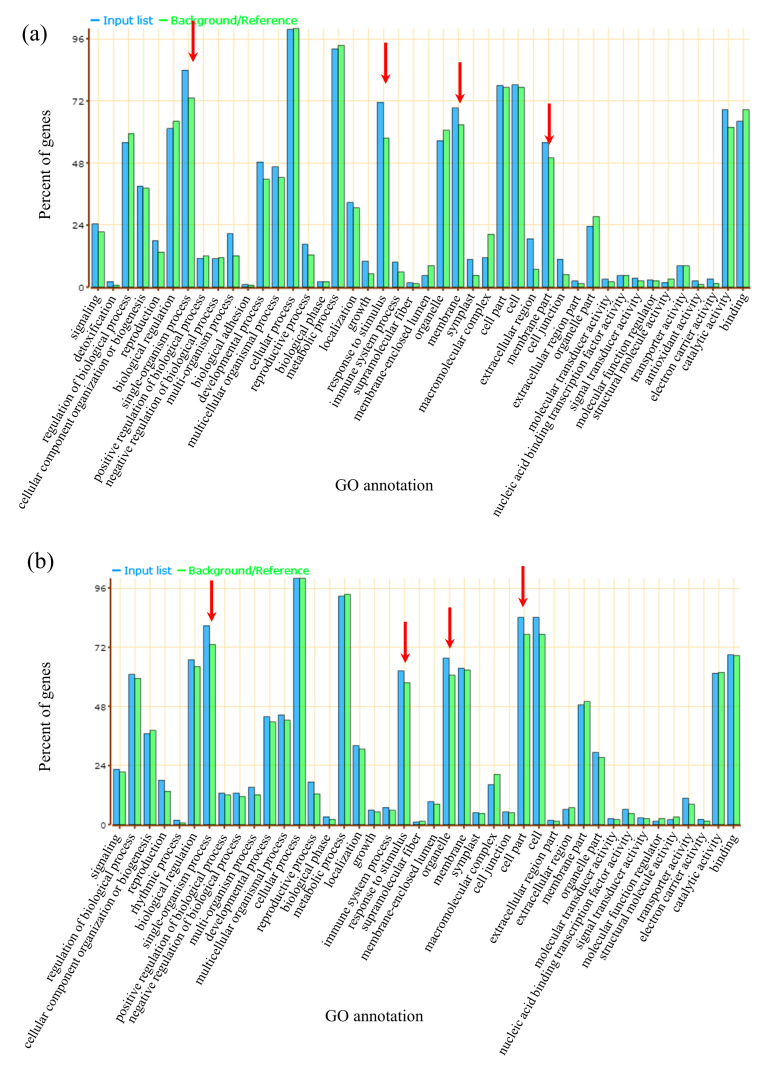
GO enrichment analysis of L282′s specifically expressed DEGs with upregulated (**a**) and downregulated (**b**) expressions at low temperature.

A GO analysis was performed to detect the functional pathways of the sDEGs (Table S4). The enriched categories of upregulated sDEGs were primarily associated with single-organism metabolic processes, the response to stimulus, and membrane and catalytic activity (Figure 7). Similarly, the downregulated sDEGs were also assigned to single-organism metabolic processes and cellular processes (Figure 7). This indicated that these categories may contribute to the low-temperature tolerance concerning seed germination of the inbred line L282. 

**Figure 8:**
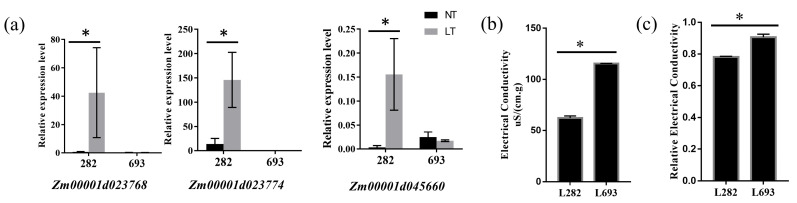
The membrane category was closely related to low-temperature tolerance in the inbred line L282. (**a**) qRT-PCR analysis of three sDEGs related to the plasma membrane system; (**b**,**c**) the comparison of electric conductivity (**b**) and relative electric conductivity (**c**) in L282 and L693 at low temperature. The asterisks indicate significant difference at *p* < 0.05 (n = 3).

At low temperature, the plasma membrane system plays a crucial role in the response to cold stress [23]. In our study, the membrane category was enriched according to the GO analysis and 586 genes were specially upregulated in L282 at a low temperature (Table S4). Three genes, which are related to lipid metabolism membranes (*Zm00001d023768*, *Zm00001d023774* and *Zm00001d045660*), were significantly upregulated in L282 at a low temperature, which was verified by qRT-PCR analysis (Figure 8a). Electric conductivity is an important index used to measure membrane permeability [24]. The electric conductivity and relative electric conductivity were both significantly lower in L282 than that in L693 at a low temperature (Figure 8b,c), indicating that the membrane of L282 experienced less damage, which is consistent with its strong tolerance. Taken together, this indicated that membrane genes may contribute to the low-temperature tolerance in the inbred line L282.

## 3. Discussion

The absence of starch accumulation has resulted in lower seed vigor, which is a major problem with respect to the seed production of sweet corn, particularly in super-sweet corn (*sh2*) [1]. Although it is caused by the *sh2* mutation, the seed vigor properties of different *sh2* sweet corn varieties show considerable differences [12]; however, the underlying mechanism behind this is unclear. 

### 3.1. Improving the Germination Capacity of Sweet Corn Seeds at Low Temperature Is Crucial for Sweet Corn Production

Seed quality is the main factor affecting the emergence of seedlings in crop production, and this parameter is mostly reflected by seed vigor [25]. Seed vigor is a comprehensive concept which refers to a seed’s emergence capacity in a wide range of environments [26]. At present, field corn with high vigor can be sown with single-seed precision, which has greatly improved production efficiency and saved labor in recent years [27]. In contrast, the seed vigor of sweet corn is typically low due to its low starch content [1]. To obtain the optimal population density, sweet corn is usually planted with 4–5 seeds per hole, followed by hand thinning at the 3-leaves-stage, which is very time-consuming and laborious [1,2,11]. Therefore, the improvement of seed vigor is crucial for sweet corn production.

Low-temperature stress is one of the main environmental factors limiting crop production [16]. Maize, whose origins lie in tropical and subtropical regions, is extremely sensitive to cold stress during the seed germination stage, which consistently causes delayed germination and reduces the seedling emergence rate [28]. Compared with field corn, the germination capacity of sweet corn is usually lower at low temperatures [1]. Adding salicylic acid or fungi to sweet corn seeds as a coating material can improve a certain degree of their germination capacity at low temperatures [29,30]. Meanwhile, germination capacity is significantly different among different sweet corn lines, implying that there are genetic factors controlling germination capacity [1]. Hence, the screening of cold-tolerance lines and the cloning of low-temperature-tolerant genes constitute an important technique for improving seeds’ germination capacity [1]. In this study, we screened two inbred sweet corn lines, which had the same germination capacity at a normal temperature but showed significantly different germination capacities at low temperatures (Figure 1). Owing to these characteristics, they were excellent materials with which to analyze the differences in the molecular mechanisms of sweet corn at low temperatures.

### 3.2. Transcriptome Profiling with Respect to Sweet Corn Kernels’ Response to Low Temperature

Low temperatures typically cause damage to the membrane system, cell dehydration, ROS accumulation, and protein denaturation [16]. At the molecular level, dynamic changes in transcription levels contribute to the adaptability of plants to environmental changes during seed germination [31]. In addition, genes involved in protein degradation, lipid-related activities, transport, redox balance, and hormone response can also improve cold tolerance in field corn [32,33]. RNA-seq technology has recently been used to study global changes at the transcript level in field corn. Li et al. (2021) used two freeze-tolerant and freeze-sensitive lines each to perform RNA-seq analysis at the seedling stage and found 948 cold-stress-contributable DEGs that were associated with binding functions, protein kinase activity, and peptidase activity [34]. Li et al. (2022) used a low-temperature-tolerant line, L220, and its introgressed lines to reveal the cell division process and plasma membrane-related categories associated with low-temperature germination [21]. 

Compared with field corn, there are few studies on the low-temperature tolerance of sweet corn using transcriptome analysis. In this study, we conducted a transcriptome analysis of seed embryos at the germination stage under normal- and low-temperature conditions to identify the DEGs involved in the response to low temperatures. Of the identified cold-germination-responsive DEGs, 830 (452 upregulated and 378 downregulated) common DEGs (cDEGs) were shared by L282 and L693, which were associated with microtubule-based processes, histone H3-K9 modification, single-organism cellular processes, carbohydrate-metabolic processes, protein binding, and so on (Figure 3 and Figure 4). In addition, we also identified 3096 (2199 upregulated and 897 downregulated) cold-germination-responsive sDEGs from the cold-tolerance line L282 (Figure 5). These sDEGs were primarily associated with single-organism metabolic processes, the response to stimulus, and membrane and catalytic activity (Figure 6). These biological processes of cDEGs and sDEGs were partially consistent with the results of similar studies conducted on field corn, indicating that there is similarity between field corn and sweet corn with respect to their responses to low temperature.

### 3.3. The Genes Related to Plasma Membrane Contribute to Low-Temperature Tolerance of sh2 Sweet Corn

The plasma membrane is an essential component in cells; it can separate internal and external spaces and plays an important role in the exchange of metabolites and signal transduction [35,36,37]. Low temperatures can inactivate the functions of the plasma membrane, including destroying its lipid bilayer structure and negating its transport activities as well as basic metabolism [38,39,40]. To cope with low-temperature stress, plant membranes have evolved a variety of adaptive mechanisms, including changing their lipid composition and increasing sugar and soluble protein content [41,42,43]. In this process, plasma membrane proteins and lipids have been well described [41,42,43]. 

The *SWEET* families are primary carbohydrate transporters in the plasma membrane that play roles in phloem loading and organogenesis [44,45]. In recent years, the *SWEET* family has also been reported to have a potential capacity for stress tolerance through controlling sugar concentrations [46,47,48]. In *Arabidopsis*, the overexpression of *AtSWEET16* not only facilitated cold adaption but also promoted seed germination [49]. The heterologous expression of the *HfSWEET17* gene led to higher cold tolerance than that of wild-type tobacco [50]. In this study, eight *SWEET* genes were found to be significantly upregulated in cold-tolerance line L282, which may represent potential candidate genes that contribute to the low-temperature tolerance of *sh2* sweet corn.

## 4. Materials and Methods

### 4.1. Plant Materials and Trait Evaluation

The *sh2* sweet corn inbred lines L282 and L693 were bred in our lab. Their seeds were harvested and stored in a controlled storage facility at 4 °C. 

For the standard germination test, 50 seeds were surface sterilized for 5 min in 0.1% sodium hypochlorite, rinsed three times with distilled water, and then sown in germination paper (Anchor Ltd., St. Paul, MN, USA). The paper was vertically rolled in a sealed plastic bag, and the paper rolls were cultured in a 25 °C chamber under a 16 h/8 h light/dark photoperiod. 

For the cold test, 50 seeds were surface-sterilized for 5 min in 0.1% sodium hypochlorite and rinsed three times with distilled water. The seeds were soaked in water at 4 °C for 3 days and then dried again to the normal moisture content. Finally, the seeds were subjected to standard germination test mentioned above.

The SL and RL were measured using a ruler at 7 days after sowing. The SW and RW were measured at the same time, and the seedlings were dried at 85 °C to a constant weight. For each trait’s evaluation, an average of 10 seedlings served as the trait value for each line. All germination tests were repeated three to four times.

### 4.2. RNA Extraction and Sequencing

Twenty-four hours after seeds were subjected to standard germination and cold germination tests, germinated embryos were collected for RNA sequencing, with two replicates for each inbred line. Total RNA was extracted using the RNeasy Plant Mini Kit (Qiagen, Hilden, Germany). The RNA concentration and quality were checked using the NanoDrop ND-2000 (Thermo Scientific, New York, USA). mRNAs were isolated using oligo (dT) magnetic beads (Illumina, San Diego, CA, USA). RNA fragmentation and PCR amplification were performed according to the RNA-seq protocol [51]. cDNA libraries were sequenced with a read length of 150 bp (paired-end) using DNBSEQ-T7 (Table S5) (Annoroad Gene Technology, Beijing, China). 

### 4.3. Sequence Data Analysis

All sequenced reads from each sample were aligned to the maize B73 reference genome (AGPv4) using HISAT2 [52]. The number of mapped reads for each gene was counted from the unique mapping reads using HTSeq-count version 1.92.2 [53]. The number of raw reads was used to calculate the FPKM values using the following formula: FPKM = read counts / (mapped reads (Millions) × exon length (KB)). A gene was considered an expressed gene if the FPKM was equal to or more than 1 in at least one sample. DEGs were identified using the DESeq2 R package with |log2fold change| ≥1 and FDR-adjusted *p*-value < 0.05 [54]. All expressed genes from different samples were submitted for PCA calculation in the R software by using the prcomp function with default settings [55].

### 4.4. Cluster Analysis and Functional Annotation Enrichment Analysis

Hierarchical clustering was performed using the hclust function in R with settings corresponding to the ward.D method. For functional annotation enrichment analysis, the agriGO online website (http://systemsbiology.cau.edu.cn/agriGOv2/, accessed on 3 July 2017) was used to perform gene ontology analysis under threshold of FDR < 0.05. 

### 4.5. Quantitative RT-PCR Analysis

First-strand cDNA was synthesized using the HiScript III 1st Strand cDNA Synthesis Kit (Vazyme, Nanjing, China) with total RNA as template. Quantitative RT-PCR was performed using Taq Pro Universal SYBR qPCR Master Mix (Vazyme) in triplicate on the Q6 Real-Time PCR System (Applied Biosystems, Foster City, CA, USA). Relative quantification was performed using the 2^−Δ^Ct method, and the *ACTIN* gene (*Zm00001d010159*) was used as control. The primers used in qRT-PCR analysis are listed in Table S6.

### 4.6. Electric Conductivity Measurement

Electrical conductivity (EC) was measured using a DDSJ-306A conductivity meter (Shanghai Precision & Scientific instrument Co., Ltd., Shanghai, China). For each line, 60 seeds were randomly selected and divided into 3 replicates. Each replicate of 20 seeds was weighed and then washed with deionized water thrice. The seeds were dried with filter paper and placed in 250 mL of deionized water. Then, EC was measured and recorded as initial EC *d*1. The seeds were soaked in water at 4 °C for 3 days; then, the EC was measured and recorded as *d*2. The final EC (μS cm^–1^/g^–1^) = (*d*2 *− d*1) /seed weight. Then, the solution (containing the seeds) was boiled for 20 min and cooled to room temperature. The solution was restored to 250 mL, and the EC was measured and recorded as *d*3. The relative EC = (*d*2 *− d*1)/(*d*3 *− d*1) × 100%.

## 5. Conclusions

We screened two inbred lines of sweet corn differing in terms of low-temperature germination capacity and conducted a transcriptome analysis to identify the genes involved in their seed germination capacity in response to low temperatures. We found that the specially expressed genes in the cold germination-tolerant line L282 were primarily related to the plasma membrane and oxygen-containing compounds, constituting a result that was partially consistent with the results of similar studies conducted on field corn. The results indicate that improving the universal low-temperature tolerance genes in field corn, such as plasma membrane-encoding genes, could effectively compensate for the low tolerance caused by starch reduction in the breeding of sweet corn. This study lays a foundation for determining and cloning key genes affecting the germination of sweet corn seeds under low-temperature conditions. 

## Figures and Tables

**Table 1:** Summary of transcriptome sequencing.

Sample Name	Line	Germination Condition	Rep	Total Reads	Rate of Total Mapped Reads (%)	Rate of Uniquely Mapped Reads (%)	Num. of Expressed Genes	Rate of Expressed Genes (%)
L282	L282	Normal	1	22,051,805	88.19%	77.64%	20,346	51.39%
			2	37,564,993	88.85%	78.52%	19,998	50.51%
L693	L693	Normal	1	23,130,884	88.83%	78.75%	20,143	50.88%
			2	23,854,290	90.11%	80.55%	20,778	52.48%
L282C	L282	Cold	1	22,230,484	89.10%	80.10%	21,045	53.16%
			2	21,280,625	88.62%	78.72%	21,067	53.21%
L693C	L693	Cold	1	22,958,482	89.93%	73.93%	21,161	53.45%
			2	35,891,174	89.21%	79.68%	21,219	53.60%

**Table 2:** L282′s specially expressed genes related to carbohydrate metabolism and transport at low temperature.

Gene	log2|FC| in L282C	FDR	log2|FC| in L693C	FDR	Annotation
*Zm00001d025943*	3.80	0.03	NA	NA	fructofuranosidases
*Zm00001d014866*	1.27	0.03	NA	NA	glycosyl transferase
*Zm00001d031303*	1.22	0.03	NA	NA	raffinose synthases
*Zm00001d037480*	1.95	0.01	NA	NA	raffinose synthases
*Zm00001d017502*	2.78	3.00 × 10^−04^	NA	NA	trehalose 6-phosphate phosphatase
*Zm00001d029371*	1.06	0.01	NA	NA	trehalose 6-phosphate phosphatase
*ZmSWEET4* *c*	3.10	3.25 × 10^−05^	NA	NA	sugar export transporter
*ZmSWEET1a*	1.07	7.49 × 10^−06^	NA	NA	sugar export transporter
*ZmSWEET4b*	1.75	1.73 × 10^−10^	NA	NA	sugar export transporter
*ZmSWEET13a*	1.39	2.16 × 10^−05^	NA	NA	sugar export transporter
*ZmSWEET13b*	1.22	2.79 × 10^−04^	NA	NA	sugar export transporter
*ZmSWEET14a*	1.50	1.56 × 10^−06^	NA	NA	sugar export transporter
*ZmSWEET14b*	1.83	6.21 × 10^−05^	NA	NA	sugar export transporter
*ZmSWEET17b*	3.44	3.20 × 10^−05^	NA	NA	sugar export transporter

## Data Availability

The data that support the findings of this study are available at https://ngdc.cncb.ac.cn/gsa/ (accessed on 20 December 2022) with accession number PRJCA012887.

## Supplementary Materials

The following supporting information can be downloaded at: https://www.mdpi.com/article/10.3390/plants12010159/s1, Table S1: common DEGs in L282 and L693 at low temperature; Table S2: GO analysis of common DEGs; Table S3: specially expressed DEGs in L282 at low temperature; Table S4: GO analysis of specially expressed DEGs in L282 at low temperature; Table S5: Summary of RNA-Seq; Table S6: primer for qRT-PCR.

## References

[1] Revilla P., Anibas C.M., Tracy W.F. (2021). Sweet corn research around the world 2015–2020. Agronomy.

[2] Hu Y., Colantonio V., Müller B.S.F., Leach K.A., Nanni A., Finegan C., Wang B., Baseggio M., Newton C.J., Juhl E.M. (2021). Genome assembly and population genomic analysis provide insights into the evolution of modern sweet corn. Nat. Commun..

[3] Tracy W.F., Shuler S.L., Dodson-Swenson H. (2019). The use of endosperm genes for sweet corn improvement: A review of developments in endosperm genes in sweet corn since the seminal publication in Plant Breeding Reviews, Volume 1, by Charles Boyer and Jack Shannon (1984). Plant Breed. Rev..

[4] (2015). The Report of the Dietary Guidelines Advisory Committee on the Dietary Guidelines for Americans, 2015, to the Secretary of Agriculture and the Secretary of Health and Human Services.

[5] Boyer C.D., Shannon J.C. (1984). The use of endosperm genes for sweet corn improvement. Plant Breed. Rev..

[6] Hannah L.C., Nelson O.E. (1976). Characterization of ADP-glucose pyrophosphorylase from *shrunken-2* and *brittle-2* mutants of maize. Biochem. Genet..

[7] Creech R.G. (1965). Genetic control of carbohydrate synthesis in maize endosperm. Genetics.

[8] Garwood D.L., McArdle F.J., Vanderslice S.F., Shannon J.C. (1976). Postharvest carbohydrate transformations and processed quality of high sugar maize genotypes. J. Am. Soc. Hortic. Sci..

[9] Carey E.E., Rhodes A.M., Dickinson D.B. (1982). Post-harvest levels of sugars and sorbitol in sugary enhancer (*su se*) and sugary (*su Se*) maize. Hortic. Sci..

[10] Juvik J.A., Yousef G.G., Han T., Tadmor Y., Azanza F., Tracy W.F., Barzur A., Rocheford T.R. (2003). QTL influencing kernel chemical composition and seedling stand establishment in sweet corn with the *shrunken2* and *sugary enhancer1* endosperm mutations. J. Am. Soc. Hortic. Sci..

[11] Douglass S.K., Juvik J.A., Splittstoesser W.E. (1993). Sweet corn seedling emergence and variation in kernel carbohydrates reserves. Seed Sci. Technol..

[12] Pairochteerakul P., Jothityangkoon D., Ketthaisong D., Simla S., Lertrat K., Suriharn B. (2018). Seed germination in relation to total sugar and starch in endosperm mutant of sweet corn genotypes. Agronomy.

[13] Juvik J.A., Jangulo M.C., Headrick J.M., Pataky J.K., Tracy W.F. (1993). Changes in characteristics of kernels in a population of *shrunken-2* maize selected for improved field emergence and increased kernel weight. J. Am. Soc. Hortic. Sci..

[14] Lertrat K., Pulam T. (2007). Breeding for increased sweetness in sweet corn. Int. J. Plant Breed..

[15] Gong F., Yang L., Tai F., Hu X., Wang W. (2014). “Omics” of maize stress response for sustainable food production: Opportunities and challenges. OMICS.

[16] Ding Y.L., Shi Y.T., Yang S.H. (2019). Advances and challenges in uncovering cold tolerance regulatory mechanisms in plants. New Phytol..

[17] Kong X.P., Pan J.W., Zhang M.Y., Xing X., Zhou Y., Liu Y., Li D.P., Li D.Q. (2011). *ZmMKK4*, a novel group C mitogen-activated protein kinase kinase in maize *(Zea mays*), confers salt and cold tolerance in transgenic Arabidopsis. Plant Cell Environ..

[18] Jiang H.F., Shi Y.T., Liu J.Y., Li Z., Fu D.Y., Wu S.F., Li M.Z., Yang Z.J., Shi Y.L., Lai J.S. (2022). Natural polymorphism of *ZmICE1* contributes to amino acid metabolism that impacts cold tolerance in maize. Nat. Plants.

[19] Li Z.Y., Fu D.Y., Wang X., Zeng R., Zhang X., Tian J.G., Zhang S.S., Yang X.H., Tian F., Lai J.S. (2022). The transcription factor *bZIP68* negatively regulates cold tolerance in maize. Plant Cell.

[20] Zeng R., Li Z.Y., Shi Y.T., Fu D.Y., Pan L., Cheng J.K., Jiang C.F., Yang S.H. (2021). Natural variation in a type—A response regulator confers maize chilling tolerance. Nat. Commun..

[21] Li X.H., Hu H.R., Hu X.M., Wang G.H., Du X.M., Li L., Wang F., Fu J.J., Wang G.H., Wang J.H. (2022). Transcriptome analysis of near-isogenic lines provides novel insights into genes associated with seed low-temperature germination ability in Maize (*Zea mays* L.). Plants.

[22] Mao J.H., Yu Y.T., Yang J., Li G.K., Li C.Y., Qi X.T., Wen T.X., Hu J.G. (2017). Comparative transcriptome analysis of sweet corn seedlings under low-temperature stress. Crop J..

[23] Steponkus P.L. (1984). Role of the plasma membrane in freezing injury and cold acclimation. Annu. Rev. Plant Physiol..

[24] Gu R.L., Huang R., Jia G.Y., Yuan Z.P., Ren L.S., Li L., Wang J.H. (2019). Effect of mechanical threshing on damage and vigor of maize seed threshed at different moisture contents. J. Integr. Agric..

[25] Rajjou L., Duval M., Gallardo K., Catusse J., Bally J., Job C., Job D. (2012). Seed germination and vigor. Annu. Rev. Plant Biol..

[26] Finch-Savage W.E., Bassel G.W. (2016). Seed vigor and crop establishment: Extending performance beyond adaptation. J. Exp. Bot..

[27] Kuş E. (2021). Evaluation of some operational parameters of a vacuum single-seed planter in maize sowing. J. Agric. Sci..

[28] Revilla P., Malvar R.A., Cartea M.E., Butrón A., Ordás A. (2000). Inheritance of cold tolerance at emergence and during early season growth in maize. Crop Sci..

[29] Gao Y., Pan S.S., Guo G.Y., Gu Q.Q., Pan R.H., Guan Y.J., Hu J. (2020). Preparation of a thermoresponsive maize seed coating agent using polymer hydrogel for chilling resistance and anti-counterfeiting. Prog. Org. Coat..

[30] Douds D.D., Wilson D.O., Seidel R., Ziegler-Ulsh C. (2016). A method to minimize the time needed for formation of mycorrhizas in sweet corn seedlings for outplanting using AM fungus inoculum. produced on-farm. Sci. Hortic..

[31] Fowler S., Thomashow M.F. (2002). Arabidopsis transcriptome profiling indicates that multiple regulatory pathways are activated during cold acclimation in addition to the CBF cold response pathway. Plant Cell.

[32] Ma Q., Dai X., Xu Y., Guo J., Liu Y., Chen N., Xiao J., Zhang D., Xu Z., Zhang X. (2009). Enhanced tolerance to chilling stress in *OsMYB3R-2* transgenic rice is mediated by alteration in cell cycle and ectopic expression of stress genes. Plant Physiol..

[33] Die J.V., Arora R., Rowland L.J. (2016). Global patterns of protein abundance during the development of cold hardiness in *Blueberry*. Environ. Exp. Bot..

[34] Li H., Yue H., Xie J., Bu J., Li L., Xin X., Zhao Y., Zhang H., Yang L., Wang J. (2021). Transcriptomic profiling of the high-vigour maize (*Zea mays* L.) hybrid variety response to cold and drought stresses during seed germination. Sci. Rep..

[35] Maxfield F.R. (2002). Plasma membrane microdomains. Curr. Opin. Cell Biol..

[36] de la Serna J.B., Schütz G.J., Eggeling C., Cebecauer M. (2016). There is no simple model of the plasma membrane organization. Front. Cell Dev. Biol..

[37] Grecco H.E., Schmick M., Bastiaens P.I.H. (2011). Signaling from the living plasma membrane. Cell.

[38] Uemura M., Tominaga Y., Nakagawara C., Shigematsu S., Minami A., Kawamura Y. (2006). Responses of the plasma membrane to low temperatures. Physiol. Plant..

[39] Kawamura Y., Uemura M. (2003). Mass spectrometric approach for identifying putative plasma membrane proteins of *Arabidopsis* leaves associated with cold acclimation. Plant J..

[40] Minami A., Fujiwara M., Furuto A., Fukao Y., Yamashita T., Kamo M., Kawamura Y., Uemura M. (2009). Alterations in detergent-resistant plasma membrane microdomains in *Arabidopsis thaliana* during cold acclimation. Plant Cell Physiol..

[41] Rahman A., Kawamura Y., Maeshima M., Rahman A., Uemura M. (2020). Plasma membrane aquaporin members PIPs act in concert to regulate cold acclimation and freezing tolerance responses in *Arabidopsis thaliana*. Plant Cell Physiol..

[42] Hirano S., Sasaki K., Osaki Y., Tahara K., Takahashi H., Takemiya A., Kodama Y. (2022). The localization of phototropin to the plasma membrane defines a cold-sensing compartment in *Marchantia polymorpha*. PNAS Nexus.

[43] Guo X., Xu H., Chong K. (2017). Cold signal shuttles from membrane to nucleus. Mol. Cell.

[44] Eom J., Chen L., Sosso D., Julius B.T., Lin I.W., Qu X., Braun D., Frommer W. (2015). SWEETs, transporters for intracellular and intercellular sugar translocation. Curr. Opin. Plant Biol..

[45] Julius B., Leach K., Tran T., Mertz R., Braun D. (2017). Sugar transporters in plants: New insights and discoveries. Plant Cell Physiol..

[46] Phukan U., Jeena G.S., Tripathi V., Shukla R.K. (2018). MaRAP2-4, a waterlogging-responsive ERF from Mentha, regulates bidirectional sugar transporter *AtSWEET10* to modulate stress response in *Arabidopsis*. Plant Biotechnol. J..

[47] Lu J., Sun M., Ma Q., Kang H., Liu Y., Hao Y., You C. (2019). *MdSWEET17*, a sugar transporter in apple, enhances drought tolerance in tomato. J. Integr. Agric..

[48] Wang L., Yao L., Hao X., Li N., Qian W., Yue C., Ding C., Zeng J., Yang Y., Wang X. (2018). Tea plant SWEET transporters: Expression profiling, sugar transport, and the involvement of *CsSWEET16* in Modifying Cold Tolerance in *Arabidopsis*. Plant Mol. Biol..

[49] Klemens P.A.W., Patzke K., Deitmer J., Spinner L., Le Hir R., Bellini C., Bedu M., Chardon F., Krapp A., Neuhaus H.E. (2013). Overexpression of the vacuolar sugar Carrier *AtSWEET16* modifies germination, growth, and stress tolerance in *Arabidopsis*. Plant Physiol..

[50] Huang D., Chen Y., Liu X., Ni D., Bai L., Qin Q. (2022). Genome-wide identification and expression analysis of the *SWEET* gene family in daylily (*Hemerocallis fulva*) and functional analysis of *HfSWEET17* in response to cold stress. BMC Plant Biol..

[51] Nagalakshmi U., Waern K., Snyder M. (2010). RNA-Seq: A method for comprehensive transcriptome analysis. Curr. Protoc. Mol. Biol..

[52] Trapnell C., Pachter L., Salzberg S.L. (2009). TopHat: Discovering splice junctions with RNA-Seq. Bioinformatics.

[53] Anders S., Pyl P.T., Huber W. (2015). HTSeq—A Python framework to work with high-throughput sequencing data. Bioinformatics.

[54] Love M., Huber W., Anders S. (2014). Moderated estimation of fold change and dispersion for RNA-seq data with DESeq2. Genome Biol..

[55] R Development Core Team (2012). R: A Language and Environment for Statistical Computing.

